# Age Macular Degeneration: Etiology, Prevention, Individualized Therapies, Cell Therapy, and Tissue Engineering

**DOI:** 10.1155/2014/287893

**Published:** 2014-03-23

**Authors:** Alfredo García-Layana, Gabriele Thumann, Jürgen Groll

**Affiliations:** ^1^Departamento de Oftalmología, Facultad de Medicina, Clinica Universidad Navarra, Avda Pio XII, 36, 31008 Pamplona, Spain; ^2^Department of Ophthalmology, University Hospitals of Geneva, Rue Alcide-Jentzer 22, 1211 Geneva, Switzerland; ^3^Department of Functional Materials in Medicine and Dentistry, University of Würzburg, Pleicherwall 2, 97070 Würzburg, Germany

Age-related macular degeneration (AMD) is the most common cause of irreversible visual loss in the western world and constitutes one of the main socioeconomical health issues worldwide. AMD is a complex multifactorial disease with an uncertain etiology associated with genetic and environmental risk factors. The majority of patients present with a slowly developing “dry” atrophic form of AMD, but about 10% of patients will develop a rapidly progressing “wet” form with choroidal neovascular membrane.

This issue on AMD compiles 8 exciting manuscripts that include meticulously performed reviews of the currently available literature and original clinical and experimental studies.

It is well-believed that the incidence of AMD and its progression to advanced stages may be reduced by controlling modifiable environmental risk factors. As a result, several groups have studied the effects of several interventions in clinical practice.

Among them, cigarette smoking is a proven risk factor for AMD. The S. Velilla et al. review summarizes the epidemiological studies evaluating the association between smoking and AMD, the mechanisms through which smoking induces damage to the chorioretinal tissues, and the relevance of advising patients to quit smoking for their visual health.

Micronutrient supplementation enhances antioxidant defense and healthy eyes and might prevent or retard AMD. The role of nutritional supplements on AMD prevention is addressed in two manuscripts. M. D. Pinazo-Durán et al. review the proposed pathogenic mechanisms of AMD, as well as the role of antioxidants and omega 3 fatty acids supplements in AMD prevention. This paper is accompanied by X. Xu et al.'s paper that explores the protective effects of Fructus lycii ethanol extract and its active components lutein/zeaxanthin* in vitro* and* in vivo* AMD mice model.

P. Fernández-Robredo et al. review the evolution of AMD treatment and the limitations of the current therapies as well as the socioeconomic impact of AMD. There is currently no cure available for AMD, and even palliative treatments are rare. Treatment options show several side effects, are of high cost, and only treat the consequence, not the cause of the pathology. For this reason, many options involving cell therapy mainly based on retinal and iris pigment epithelium cells as well as stem cells are being tested. Moreover, tissue engineering strategies to design and manufacture scaffolds to mimic Bruch's membrane are very diverse and under investigation. Both alternative therapies are aimed at preventing and/or curing AMD and are reviewed herein.

The establishment of future retinal pigment epithelium (RPE) replacement therapy is partly dependent on the availability of tissue-engineered RPE cells, which may be enhanced by the development of suitable storage methods for RPE. L. Pasovic et al. show in their article that the preservation of RPE cells is critically dependent on storage temperature.

Rheopheresis is an apparently safe and effective form of membrane differential filtration for the elimination of high molecular weight proteins. J. Studnička et al.'s paper deals with those aspects showing that rheopheresis reduced drusenoid pigment epithelium detachment, improved the electroretinographic function of photoreceptors, and prevented the decline of visual acuity.

Treatment of neovascular AMD is further reviewed in two papers. K. Fang et al. identify the predictors of visual response to the bevacizumab treatment of neovascular AMD in a multicenter trial including 144 participants from the NATTB study. Baseline VA and genotype of rs10490924 were both important predictors for visual response to bevacizumab at 6 months. Finally, R. Casaroli-Marano et al. conducted an observational retrospective multicenter study of the management of patients with neovascular AMD. Achieving the visual outcomes reported in pivotal trials, early diagnosis, a proactive approach (more treating than follow-up visits), and close monitoring might be the key to successfully manage neovascular AMD.

*Alfredo García-Layana* *Alfredo García-Layana* *Gabriele Thumann* *Gabriele Thumann* *Jürgen Groll* *Jürgen Groll*

